# Supplementary material for: Timely deployment of best-in-class technologies to enable development and decarbonise construction
Source: Nat Commun. 2025 Dec 24;17:799. doi: 10.1038/s41467-025-67489-8 (PMC12824142; doi:10.1038/s41467-025-67489-8)
Supplement: Supplementary file 1 — Supplementary Information [file 41467_2025_67489_MOESM1_ESM.pdf]

Supplementary Information - Timely deployment of best in class technologies to enable development and decarbonise construction

Cyrille Dunant[1], Hisham Hafez[2], Alastair T.M. Marsh[3], Sabbie Miller[4], Martin Röck[5], Wolfram Schmidt[6], Karen Scrivener[3*], Franco Zunino[7]

[1] Engineering Department, University of Cambridge, Cambridge CB2 1PZ, UK

[2] School of Civil Engineering, University of Leeds, Leeds LS2 9JT, UK

[3] Laboratory of Construction Materials, Ecole Polytechnique Federale de Lausanne, Lausanne 1015, Switzerland

[4] Civil and Environmental Engineering, University of California, Davis, USA

[5] RISE Institute for Regenerative Spatial Systems Science, Vienna, Austria

[6] Bundesanstalt für Materialforschung und -prüfung, 12205 Berlin, Germany

[7] Department of Civil and Environmental Engineering, University of California, Berkeley, USA

* corresponding author: karen.scrivener@epfl.ch

LIST OF SUPPLEMENTARY ITEMS

Supplementary Methods – pg.3 onwards

Supplementary Tables:
Supplementary Table 1 – pg.4
Supplementary Table 2 – pg.5
Supplementary Table 3 – pg.10
Supplementary Table 4 – pg.12
Supplementary Table 5 – pg.12
Supplementary Table 6 – pg.13
Supplementary Table 7 – pg.13
Supplementary Table 8 – pg.13
Supplementary Table 9 – pg.14
Supplementary Table 10 – pg.15
Supplementary Table 11 – pg.19

Supplementary Figures:
Supplementary Figure 1 – pg.10
Supplementary Figure 2 – pg.11
Supplementary Figure 3 – pg.11
Supplementary Figure 4 – pg.19

Supplementary References – pg.21 onwards

ESTIMATE OF GLOBAL MATERIALS CONSUMPTION AND CORRESPONDING CO_2 EMISSIONS

A summary of values used for Figure 1 in the main article is given in Supplementary Table 1 below. As described in the Methods section of the main article, details around assumptions and calculations for generating the values in Supplementary Figure 1 are given in the subsequent sub-sections.

Supplementary Table 1: Summary of values for current worldwide production and emissions of cement-based materials, brick, glass, asphalt concrete, steel, aluminium, plastics, wood, plasterboard and mineral wool.

**Material**	Annual production (Mt)	Annual production (Mm^3)	Sources	Density (kg/m^3)	Sources	Proportion used in construction (%)	Sources	Annual amount used in construction (Mt)	Fossil CO_2 emissions (all uses) (Mt)	Sources	Estimated emissions intensity (kg CO_2/kg)
Cement-based materials	26,681	12,239	[1]	2,180	own calculation	100%	own calculation	26,681	2,909	[2-4]	0.11
brick	2,180	1178	[5]	1,850	[6]	100%	own calculation	2,180	510	[5]	0.23
glass	150	61	[7]	2,450	[6]	39%	[7]	59	98	[8]	0.65
asphalt concrete	2,244	966	[9]	2,323	[10]	100%	own calculation	2,244	115	[11]	0.05
cast iron and low alloy steel	1,860	248	[12]	7,500	[6]	55%	[13]	1,023	3,741	[14,15]	2.01
aluminium	63	23	[16]	2,700	[6]	24%	[17]	15	1,000	[18]	15.8
plastics	414	385	[19]	1,076	own calculation	20.4%	[19]	84	2,200	[20]	5.31
wood	3,092	4,235	[21]	730	[6]	10.9%	own calculation	337	773	[22]	0.25
plasterboard	87	135	[23]	640	[24]	100%	own calculation	87	21	[25]	0.24
mineral wool	19	190	[26]	100	[27]	100%	own calculation	19	29	[25,28]	1.53

Cement-based materials
There are three broad categories of cement-based materials:

1. Concrete (reinforced and non-reinforced)
2. Mortars mixed on site, with sub-categories of mortars to hold bricks, blocks or stones together in masonry, and coatings variously referred to as renders or plasters (however, plaster is also used for gypsum-based coatings)
3. “Concrete products” produced in factories such as blocks, pavers and tiles.

The mass of cement production worldwide was reported by United States Geological Survey (USGS) to be 4.1 Gt in the year 2019 [1]. Because the production of concrete, mortar and concrete products is highly decentralised (unlike cement production), there is no official estimate for the annual production of all cement-based materials. Therefore, to estimate all cement-based materials production, approximate global market share and cement contents were used. For market share, the breakdown between these categories varies significantly: values from Brazil [29] (40% concrete, 40% mortar, and 20% concrete products) were taken as representative of the world average, given the position of Brazil as an emerging economy. We note that individual regions will differ in values, but for the global assessment, the values reported in Brazil were used.

For concrete the cement content lies in the range 250 to 400 kg/m^3 for the strength classes which make up the majority of global concrete use [30]. For mortars, paste contents are generally higher as a consequence of the maximum aggregate size being smaller and need to apply the mortars by hand on-site, but some of the cement may be replaced by filler, 150-500 kg/m^3 is a reasonable range [31]. The cement content for concrete products varies from these other two categories due to differences tied to prefabrication processes and different performance requirements. Here, a cement content range of 250-500 kg/m^3 [32] is considered. The market share-weighted average of the midpoint values of cement content for each sub-category gives a global estimate of 335 kg/m^3 (Supplementary Table 2). Using this average cement content and the global cement production of 4.1 Gt, we estimate that in 2019, approximately 12.2 Mm^3 of cement-based materials were produced.

The physical density of cement-based materials also varies between concretes, mortars and cement-based products. Concretes and cement-based products typically have a higher range of density (2200-2600 kg/m^3 [33]) compared to mortars (1700-2000 kg/m^3 [31]). The market share-weighted average of the midpoint values of physical density for each sub-category is 2180 kg/m^3 (Supplementary Table 2). Using this average density we estimate the annual production mass of cement-based materials to be 26.7 Gt in 2019. The reinforcing steel used in most reinforced concrete is not considered in these calculations in order to avoid double-counting, as the production and use of steel is considered separately at the sectoral level in Supplementary Table 1.

Supplementary Table 2: Summary of market share, cement content and physical density for cement-based materials, and global averages for cement-based materials.

**Category**	**Market share [29]**	**Cement content (kg/m^3) [30-32]**	**Density (kg/m^3) [31,33]**
Concrete	40%	250-400	2200-2600
Site mixed mortars	40%	150-500	1700-2000
Concrete products	20%	250-500	2200-2600

Market share weighted average	n/a	335	2180

Embodied CO_2 emissions for cement (excluding electricity) in 2019 were reported by IEA[39] to be 0.58 kg CO_2 / kg cement. Electricity demand was reported as 102 kWh per metric ton of cement in 2019 [3]. Using the global emissions per kWh of electricity reported by the IEA (approximately 470 g $CO_{2\text{-eq}}$ / kWh), we estimate emissions from electricity demand for cement production (~0.048 kg $CO_{2\text{-eq}}$ / kg of cement, and we use this value to estimate CO_2 emissions for cement production). Other constituents in cement-based materials (inc. aggregates, water, admixtures) contribute additional embodied carbon; however, given the compositional diversity of cement-based materials between and within sub-categories, the relative magnitude of these contributions will vary. For concrete and concrete-based products, an approximate contribution of 88.5% of CO_2 emissions from cement, with remaining emissions coming from other constituents was used, based on [34]. Noting that emissions were calculated based on cement production and other constituents contribute an additional >10% of emissions, this additional contribution to the CO_2 emissions for cement-based materials production was included, and we estimate the approximate CO_2 emissions from "cement-based materials" as being 2.91 Gt. Dividing global CO_2 emissions by the global production mass of cement-based materials estimated above (26.7 Gt/year) gives an emissions factor of 0.11 kg.CO_2/kg.cement-based material. The estimated total anthropogenic CO_2 emissions was 45 ± 5.5 Gt.CO_2 in 2019 [35]; the above estimate therefore indicates that cement-based materials are responsible for approximately 5.8-7.4% of global anthropogenic CO_2 emissions. When discussing resource flows to construction, we assume all cement-based materials are used in construction applications.

Concrete can remove some CO_2 from the atmosphere and sequester it as calcium carbonate. The amount of CO_2 sequestered from the atmosphere is affected by several parameters, including exposure (e.g., CO_2 concentrations, relative humidity, pore saturation), mixture characteristics (e.g., amount of CH, ratio of Ca/Si in CSH, porosity), as well as exposed surface area and the duration of exposure [36-39]. It has been proposed that a substantial fraction of the CO_2 emitted during calcination can effectively be reabsorbed by recarbonation [36,37,40]. Yet the time difference between the rapid emissions from calcination followed by the decadal process of CO_2 uptake during carbonation means their net effect on cumulative radiative forcing is not a simple summation of the quantity of emissions and the quantity of uptake [36]. While this is an important area of investigation, re-carbonation is outside the scope of this work and not included in our estimates.

Brick

The annual mass production was estimated by Olsson *et al.* [5] to be 2.18 Gt/year in 2020. To calculate the annual volume production of brick, we estimate the density as being 1850 kg/m^3 (based on [6]), which then gives an annual production volume of 1.18 Mm^3/year. An estimate of the CO_2 emissions, without electricity demands, are reported by Olsson *et al.* [5], approximately 0.51 $CO_{2(eq.)}$: equivalent to an estimated global average emissions factor of 0.23 kg.CO_2/kg.brick [5]. We noted that reported values of brick production are notably sparse. There is also a substantial range between emissions associated with efficiency of various kilns and associated with different energy resources. Reports indicate that substantial fractions of global production still rely on outdated kiln technologies [5] and high-emitting energy resources, such as coal [41]. However, due to poor data quality, we note that the values used

for brick in this report are highly uncertain. As with cement-based materials, we assume all brick production is for use in construction.

Glass

Recent data for global glass production were not readily reported. Therefore, in this work, we use an annual production mass estimate of 150 Mt/year in 2014 from Westbroek *et al.* [7]. To calculate volume, the density of glass is estimated as 2450 kg/m^3 (based on [6]), giving annual volume production as 61 M m^3/year. In this work, 0.65 kg.CO_2/kg.glass was used as an emissions factor, summing contributions for direct fuel emissions (0.45 kg.CO_2/kg.glass) and process emissions from thermal decomposition of limestone and soda ash (0.2 kg.CO_2/kg.glass) as reported by the IEA in 2007 [8]. Multiplying by the global production of glass, we estimate annual global CO_2 emissions of 97.5 Mt in 2019. Herein, we estimate that approximately 39% of glass is used in construction applications [7].

Asphalt Concrete

To calculate asphalt concrete production, we assume the mass fraction of asphalt pavement is 5% bitumen and 95% aggregates. Recent bitumen statistics were not available, so a value of 112 Mt of bitumen produced in 2016 as reported by the United Nations Statistics Division [9] was used. Including the mass fraction from aggregate, this resulted in 2244 Mt of asphalt concrete. Density was assumed to be 2322.9 kg/m^3 [10], giving an estimated annual production volume of 966 Mm^3.

CO_2 emissions from asphalt concrete production were based on a report from the United States, prepared by the National Asphalt Pavement Association, which reported the emissions factor for asphalt concrete in 2019 to be between 50.2 to 52.1 kg.CO_2/tonne of asphalt concrete [11]. Herein, we take the average of these values. Applying this emissions factor to estimated annual global production mass leads to an estimate of 115.4 Mt of CO_2 emissions in 2019. We assume all asphalt concrete production is for use in construction.

Steel

The mass of steel produced in 2019, as reported by the USGS, was approximately 1860 Mt [12]. Using an average density of 7500 kg/m^3 (based on [6]), we estimated the annual volume of steel production as 248 Mm^3. An estimate of the CO_2 emissions are reported by the IEA [14], again without electricity demands, to be approximately 1.42 kg CO_2 / kg steel in 2020. We assume production technologies in 2019 and 2020 to be similar enough to apply the same emissions factor for 2019. Using global steel production values above, this emissions factor can be used to calculate the CO_2 emissions from steel as being 2.64 Gt. In addition to these direct emissions, there are numerous sources of Scope 2 emissions tied to the production of steel. Based on a report by the IEA [15], it can be estimated that the use of off-gases, along with other energy resources leads to another 1.1 Gt of CO_2 emissions. Together, we estimate that global CO_2 emissions from steel production were approximately 3.74 Gt in 2019. Dividing by steel production in that year (1.86 Gt), we estimate an average emissions factor for steel production as being 2.01 kg.CO_2/kg.steel. We note that there are numerous steel alloys, and emissions factors for different regions, furnace types, and alloys will vary notably from this estimated average. For calculations of material flows to construction, we estimate that 55% of steel is used in construction applications (Figure 1 of Cullen et al. [13]).

Aluminium
The mass of aluminium produced in 2019, as reported by the USGS, was approximately 63.2 Mt [16]. We estimate the density as being 2700 kg/m^3 (based on [6]), and use this value to calculate the annual production volume of aluminium to be 23 Mm^3. An estimate of the CO_2 emissions reported by the IEA [18], again without electricity demands, is 270 Mt in 2022. We note, the IEA indicates that these CO_2 emissions could be up to 1 Gt when Scope 2 emissions are included. Therefore, we use the 1 Gt of CO_2 emissions in our calculations to maintain a consistent scope of assessment. Dividing global production emissions by annual production mass gives an emissions factor of 15.8 kg.CO_2/ kg.aluminium. When examining different resource flows, we assume 24% of aluminium is used in construction applications [17].

Plastics
Global plastics mass production was reported by PlasticsEurope to be 368 Mt in 2019 [19]. However, the data reported from this year did not contain recycled mass, which we estimate using the approximation reported by Geyer et al. [42]. With this additional factor to account for recycled plastic mass, we estimate 414 Mt of plastics were produced in 2019.

The physical density of plastics varies between, and within, sub-categories of plastics. We calculated a global market share weighted average, using the midpoint physical density value, for the following plastic types which data was available for both market share [19] and physical density [6]: Polyethylene terephthalate, Polyethylene (low density (LD) and linear low density (LLD)), Polyethylene (high density (HD) and medium density (MD)), Polypropylene, Polystyrene, Polyvinylchloride, and Polyurethane. Combined, these plastic types together represent 76% of global production mass. The global market share weighted average physical density is 1076 kg/m^3. However, this is likely a higher-than-accurate cumulative density for plastics due to their regular application in foams and similar low-density products, for which market share value information is less easily accessible.

Global CO_2 emissions from plastics production in 2020 were estimated to be 2.2 Gt.CO_2 [20]. Dividing by the global plastics mass production in 2019 gives an estimated 5.31 kg.CO_2/kg.plastic as an average over all plastic types. In Europe, approximately 20.4% of plastics produced were used in building and construction in 2019 [19] and when estimating mass flows to construction, we apply this fraction globally.

Wood
The annual production volume of wood produced in 2019, as reported by the United Nations FAOSTAT database for "Roundwood" [21], was 4,235 million m^3. The restriction for roundwood was used, as this generally classifies wood for use in products rather than fuels. Physical density was assumed to be 730 kg/m^3 (a midpoint value of the range reported in [6]), which was then used to calculate annual production mass of 3.09 Gt/year.

Carbon accounting applied to biogenic carbon, and particularly carbon from forest products used in construction applications, can be a difficult task; this results in a range of modelling outputs driven by incompatible modelling assumptions, regional variations in forest management strategies, land use changes, and different temporal envelopes, among others [43,4466,67]. When addressing the embodied carbon from manufacturing-related GHG emissions (i.e., only energy-derived emissions from material processing and not CO_2 taken in during photosynthesis, biomass decomposition in the forest, or land-use changes), it is typically seen that these emissions commonly fall in the range of between 150-300 kg CO_2/t of timber for

softwood to close to 400-500 kg CO_2/t of engineered wood products such as CLT [454]. In this study, we use 250 kg.CO_2 /t of timber in construction as a representative global value; this is a weighted average for CLT calculated by the Institution of Structural Engineers (IStructE), based on the A1-A3 emissions reported by 11 EPDs in the European region [22]. Applying this value gives an annual emissions for timber of 773 Mt.CO_2/year. However, we note that the disposal, sequestration and potential recovery of timber at the end of life of the building can lead to widely varying outcomes, with the IStructE, for example, suggesting close to -1500 kg CO_2/t of timber for sequestration, and 1600 kg/CO_2 for the disposal [46]. Further, the dynamic factors associated with timing of CO_2 removal from the atmosphere, the period during which it remains in biomass, and the rate at which it is released (if at all), can further affect how models report net-emissions [47].

The average worldwide share of harvested roundwood used in harvested wood products (as opposed to fuels) is estimated to be 48% [43]. Within harvested wood products, the average worldwide share used in construction is estimated to be 22.7% [48]. Combining these two values, the average worldwide share of harvested roundwood used in construction products is estimated to be 10.9%.

Plasterboard

The annual global production of gypsum plasterboard in 2019 was reported to be 10,836 Mm^2/year, by area [23]. To convert this into annual production volume, a representative thickness of 12.5 mm was assumed, which is a midpoint value of the common thicknesses sold [24]. This gives an annual production volume of 135 Mm^3. To convert annual production volume to annual production mass, a representative density of 640 kg/m^3 was assumed - again, a midpoint value from common products [24]. Multiplying annual production volume by density gives an annual production mass of 87 Mt/year. An embodied carbon value of 0.238 kg.$CO_{2(eq.)}$ /kg was used, derived from the average of numerous EPDs reported in the ICE 4.0 Database [25]. Multiplying this value by the annual production mass gives the embodied carbon for annual plasterboard production of 21 Mt.$CO_{2(eq.)}$/year. The proportion of plasterboard used in construction was assumed to be 100%.

Mineral wool

Amongst types of insulation materials, mineral wool is the only material for which we deem there is sufficient data available to make a reasonable estimate of annual production quantities and emissions. The annual global production of mineral wool by mass in 2019 was reported to be 19 Mt/year [26]. To convert this into annual production volume, a representative density of 100 kg/m^3 was used – this is a midpoint value for the common range of mineral wool densities (25 – 200 kg/m^3) [27]. Dividing annual production mass by density gives an annual production volume of 190 Mm^3/year. An embodied carbon value of 1.53 kg.$CO_{2(eq.)}$ /kg was used, derived from the average of numerous EPDs reported in the ICE 4.0 Database [25]; this also matches well with the midpoint of the range established from EPD data in another study [28]. Multiplying this value by the annual production mass gives the embodied carbon for annual mineral wool production of 29 Mt.$CO_{2(eq.)}$/year. The proportion of mineral wool used in construction was assumed to be 100%.

TOP-DOWN ESTIMATION APPROACH

As described in the Methods section, certain outlier countries were excluded from the dataset. These included island nations and petro-states with atypical economic characteristics (see Supplementary Table 3). Oil-exporting countries (Kuwait, Oman, Saudi Arabia, UAE and Qatar) were outliers, due to their large transient populations, atypical consumption patterns and the large contribution of hydrocarbons to their GDP (>40%) [49].

Supplementary Table 3: Countries excluded for the purpose of calculating a master curve. The difficulty of computing the GDP of Ireland is well-documented [76].

**Country**	Qatar	Kuwait	UAE	Saudi Arabia	Oman
**Reason**	Petro-state	Petro-state	Petro-state	Petro-state	Petro-state
**Country**	Bahrain	Singapore	Bhutan	Ireland	Cyprus
**Reason**	Petro-state	City-state	Petro-state	Uncertain GDP	Tax haven

The master curves for cement (Supplementary Figure 1) and steel (Supplementary Figure 2) are shown below, with selected individual countries labelled. The master curves for both cement and steel are plotted together in Supplementary Figure 3.

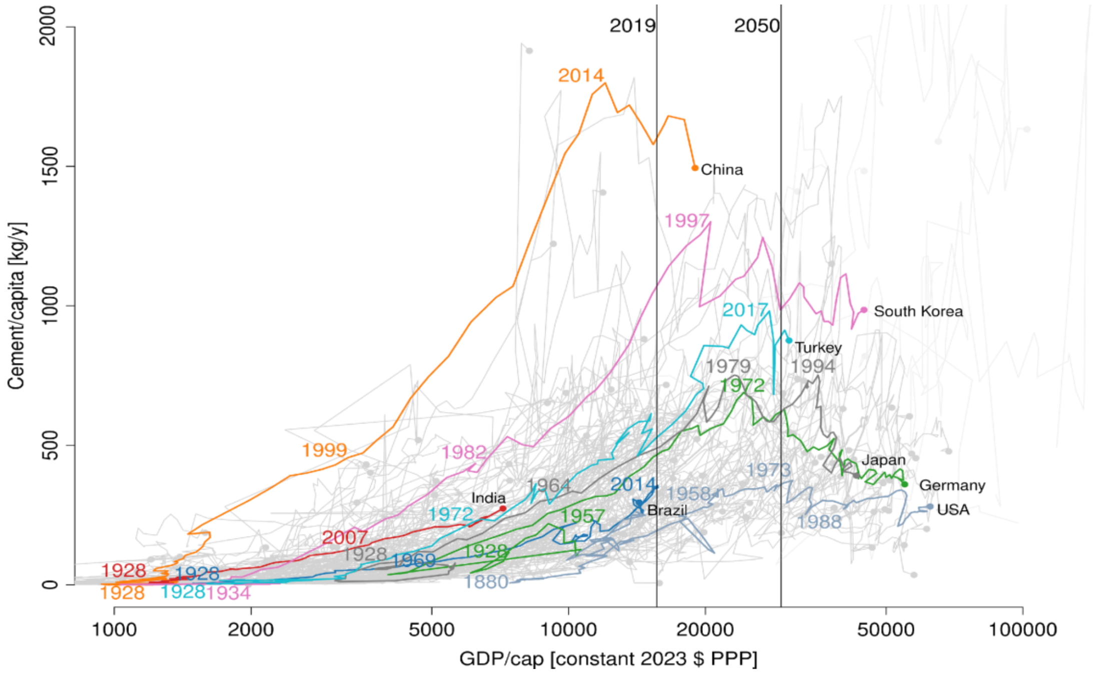

Supplementary Figure 1: Cement consumption per capita of the countries not excluded from the dataset for the purpose of calculating the master curve. Countries which at any point represented more than 2% of the global consumption are highlighted. The vertical lines mark the world's GDP/cap in 2020 and expected in 2050.

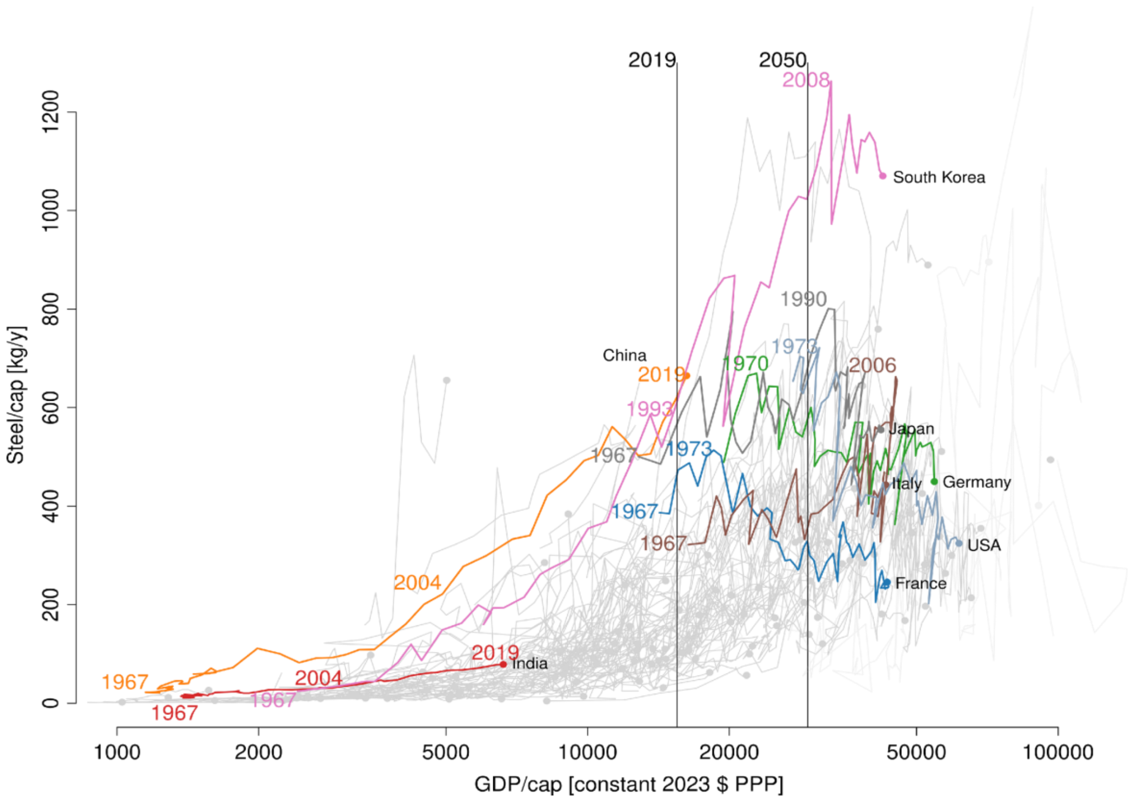

Supplementary Figure 2: Steel consumption per capita of the countries not excluded for the purpose of calculating the master curve. Countries which at any point represented more than 2% of the global consumption are highlighted. The vertical lines mark the world's GDP/cap in 2020 and expected in 2050.

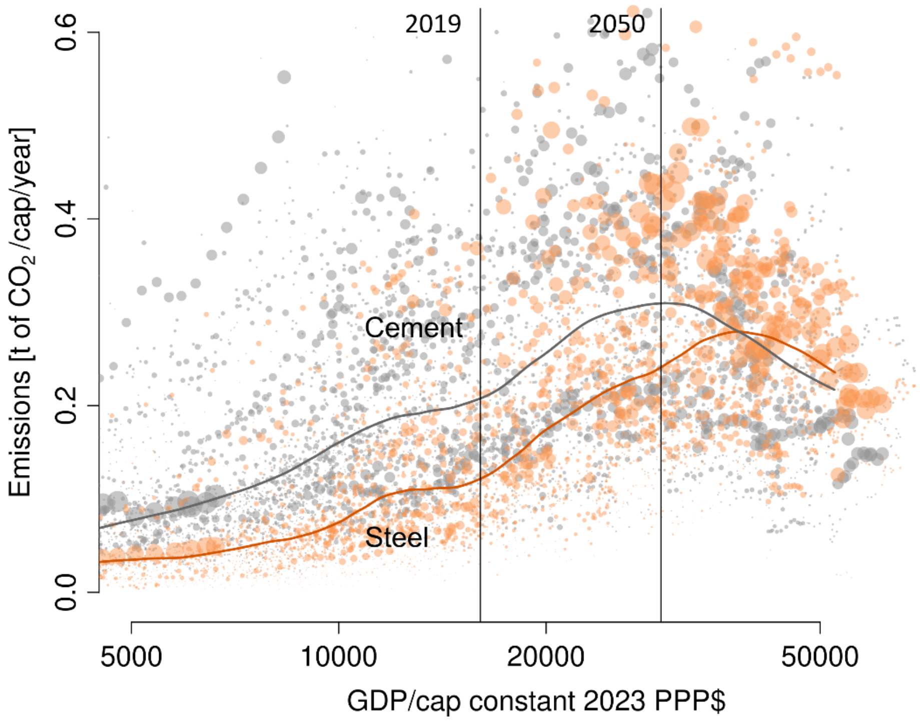

Supplementary Figure 3: Master curves of CO_2 emissions against GDP/capita for steel (orange data points) and cement (grey data points).

BOTTOM-UP ESTIMATION APPROACH

A database (Supplementary Table 4) was created to estimate the global material intensity (kg/m^2 floor area) in the current buildings stock as a weighted average of the different typologies (single unit, mid-rise and high-rise buildings), functions (commercial, industrial and residential) as well as primary material (concrete, steel or timber buildings).

Supplementary Table 4: Range of material intensity values for different materials in buildings.

Material	Material Intensity (kg/m²)																
	Min	Max	Avg	St.Dev	References												
					50	51	52	53	54	55	56	57	58	59	60	61	62
Concrete	650	1657	988	305	908	650	-	1657	931	827	829	-	863	705	1200	908	1390
Bricks	160	493	315	131	-	160	-	-	-	296	493	234	-	-	-	392	-
Timber	27	205	97	60	-	80	180	205	-	-	78.9	27	100.7	44.06	-	103	56
Steel	52	80	67	13	-	60	-	-	-	-	52	80	-	-	-	-	75

A global average was estimated for the split between concrete use in buildings and infrastructure, based on previous literature (Supplementary Table 5). Whilst there have been some data published around global material stocks in infrastructure [63], there are not yet any comprehensive datasets for global flows of construction materials into infrastructure. Future developments in this field could be used to update and improve the approach used in this study.

The estimated proportion of material use in buildings, out of total use in construction, are given for each material in Supplementary Table 6.

Supplementary Table 5: Distribution of cement and concrete between buildings and infrastructure for various world regions.

Cement material flows		Concrete material flows		Country	Source
Concrete	Mortar	Buildings	Infrastructure		
-	-	60.0%	40.0%	Iran	64
35.0%	65.0%	-	-	Brazil	65
73.0%	27.0%	65.0%	35.0%	Global	40
58.0%	42.0%	-	-	China	66
77%	23%	-	-	United Kingdom	67
80%	20%	50.0%	42.3%	Japan	68
73%	27%	53.0%	47.0%	Global	69
66.0%	*34.0%*	*57.0%*	*43.0%*	*Global average values*	

*Supplementary Table 6: Fraction of material use in buildings out of total use in construction (i.e. buildings and infrastructure). *The value here is for concrete, rather than all cement-based materials, as the Deetman et al. projections are for concrete.*

Material	Fraction of use in buildings out of total use in construction
Concrete*	38%
Bricks	100%
Timber	100%
Steel	60%

The material demand from buildings only was calculated using average material intensity values (Supplementary Table 7).

Supplementary Table 7: Range of material demand in buildings only in 2019 and 2050 for cement-based materials, bricks, timber and steel.

Material	2019			2050		
	Min.	Avg.	Max.	Min.	Avg	Max.
	Material demand in buildings (Mt/year)					
Cement-based materials	5.38	8.17	13.70	7.27	11.05	18.53
Bricks	1.32	2.61	4.08	1.79	3.52	5.51
Timber	0.22	0.80	1.70	0.30	1.09	2.29
Steel	0.43	0.55	0.66	0.58	0.75	0.89

Additional projected demand for cement-based materials and steel from infrastructure (as explained in the Methods section of the main article) was then added to estimate construction demand in 2050 (Supplementary Table 8). These estimates for total material consumption of the cement-based materials, steel, and timber using the bottom-up approach were presented for 2019 and 2050 in Figure 3A in the main article.

Supplementary Table 8: Range of material demand in total construction in 2019 and 2050 for cement-based materials*, bricks, timber and steel.*

Material	2019			2050		
	Min.	Median	Max.	Min.	Median	Max.
	Material inflows in construction (Mt/year)					
Cement-based materials	14.29	21.72	36.43	19.33	29.37	49.27
Bricks	1.32	2.61	4.08	1.79	3.52	5.51
Timber	0.22	0.80	1.70	0.30	1.09	2.29
Steel	0.71	0.92	1.10	0.97	1.24	1.49

The estimated cumulative cement-based materials demand for 2019-2025 for each region is given in Supplementary Table 9; 2019 GDP/capita values for individual countries were the same used for the top-down analysis as described earlier, and used to generate a population-weighted average GDP/capita value for each of the 26 regions.

Supplementary Table 9: For the twenty six world regions defined in the IMAGE platform, GDP/capita values and projected cumulative cement-based materials demand from 2019-2050.

IMAGE region	GDP/capita (2019, region weighted average)	Cement-based materials total demand 2019-2050 (Gt)
Canada	$48,741.43	7.9
USA	$61,547.55	47.3
Mexico	$20,159.42	16.8
Central America	$10,595.66	9.7
Brazil	$14,600.88	18.1
Rest of South America	$15,433.22	30.0
Northern Africa	$10,721.72	18.2
Western Africa	$3,058.02	40.9
Eastern Africa	$2,484.57	18.1
South Africa	$12,583.07	7.5
Western Europe	$48,085.73	56.2
Central Europe	$30,523.25	13.4
Turkey	$28,271.44	11.4
Ukraine region	$13,989.83	4.5
Central Asia	$11,927.92	5.6
Russia region	$25,774.02	16.5
Middle East	$19,366.42	31.3
India	$6,619.19	110.2
Korea region	$42,399.98	7.8
China region	$17,038.50	221.0
Southeastern Asia	$12,485.36	33.7
Indonesia region	$11,552.88	18.3
Japan	$42,021.15	14.6
Oceania	$48,138.42	6.1
Rest of South Asia	$4,719.44	30.0
Rest of Southern Africa	$3,636.63	10.8

COMPARISON OF THE POTENTIAL OF MITIGATION APPROACHES TO LOWER CO_2 EMISSIONS FROM CONSTRUCTION MATERIALS

The carbon mitigation values for different strategies, as presented in Figure 4 in the main article, were obtained via applying the cumulative reduction factors for the different strategies (Supplementary Table 10) to the ‘top-down’ extrapolated material demand using the master curve (Supplementary Figure 3). Upper bounds and lower bounds for CO_2 emissions of global cement and steel production in 2050 were estimated, assuming the most unfavourable penetration (or calculation method) and the most favourable, respectively.

The three sections of Supplementary Table 10 follow the same format. Rows “Fe factor” and “Cem factor” state the proportional reductions in emissions for each material, for each individual intervention (not cumulative). Rows “Steel”, “Cement”, and “Timber” state the net

emissions for each material (Gt.CO_2/year), considering the cumulative effect of strategies in each column. Rows "Substitution", "Efficiency", and "Process" state the cumulative emissions savings (Gt.CO_2/year) from each category of strategy, across both cement and steel. "Substitution" strategies are: timber substitution, new SCMs, and CEM recycling. "Efficiency" strategies are: better design, better concrete. "Process" strategies are: EAF/DRI.

Estimating mitigation potential to 2050 requires judgements about the materials or emissions reductions achievable for a given unit of structure or material. Given the subjective nature of such assumptions, it is important to describe the rationale and evidence base behind these judgments when estimating emission mitigation potential [70]. In each following sub-section, a detailed description is given of how the mitigation potential for each strategy, and the upper and lower bounds, were estimated.

Supplementary Table 10: Base, upper bound (i.e. *pessimistic*) *and* lower bound (i.e. *optimistic*) *value factors used to construct Figure 4 in the main article, which shows the potential for decarbonisation of the cement and steel sectors accumulated across a range of interventions (as listed in the columns, accumulating from left to right).*

	Parameter	Initial	Timber Substitution	Better design	New SCMs	Better concrete	EAF/DRI	CEM recycling
Base values	Fe factor		0.018	0.125	0	0	0.65	0
	Cem factor		0.036	0.125	0.3	0.4-0.3-0.15	0	0.025-0.1-0.15
	Steel	0.79	0.78	0.68	0.68	0.68	0.24	0.24
	Cement	1	0.96	0.84	0.59	0.41	0.41	0.37
	Timber	0.03	0.03	0.03	0.03	0.03	0.03	0.03
	Substitution	0	0.05	0.05	0.31	0.31	0.31	0.33
	Efficiency	0	0.00	0.22	0.22	0.39	0.39	0.39
	Process	0	0.00	0.00	0.00	0.00	0.44	0.46
Upper bounds of emissions	Fe factor		0.0288	0.075	0	0	0.425	0
	Cem factor		0.0576	0.075	0.15		0	0.025
	Steel	0.79	0.77	0.71	0.71	0.71	0.41	0.41
	Cement	1	0.94	0.87	0.74	0.63	0.63	0.61
	Timber	0.06	0.10	0.08	0.08	0.08	0.08	0.08
	Substitution	0	0.04	0.04	0.18	0.18	0.18	0.18
	Efficiency	0	0.00	0.13	0.13	0.24	0.24	0.24
	Process	0	0.00	0.00	0.00	0.00	0.30	0.31
Lower bounds of emissions	Fe factor		0.0288	0.25	0	0	0.9	0
	Cem factor		0.036	0.25	0.4	0.4	0	0.15
	Steel	0.79	0.77	0.58	0.58	0.58	0.06	0.06
	Cement	1	0.96	0.72	0.43	0.26	0.26	0.22
	Timber	-0.08	-0.09	-0.07	-0.07	-0.07	-0.07	-0.07
	Substitution	0	0.07	0.07	0.36	0.36	0.36	0.38
	Efficiency	0	0.00	0.43	0.43	0.61	0.61	0.61
	Process	0	0.00	0.00	0.00	0.00	0.52	0.54

Substitution of concrete with timber and other bio-based materials

Wood is a popular choice as a structural and non-structural construction material in many regions. Wood has acquired a high appeal in recent years due to its biogenic nature, where forests are accounted for as CO_2 sinks. However, the harvesting of wood-based products is

far from being a carbon neutral process, with overall associated emissions of 2.7 to 3.2 Gt $CO_{2(eq.)}$ per year considering the supply level of 2010 [43]. Moreover, wood is only available in certain regions and in very limited amounts compared to concrete, and the amount of land available for additional forests is limited and geographically mismatched with the projections of construction material consumption up to 2050 [71]. The academic literature contains very different estimates for what quantities of bio-based material harvesting are possible: proposed upper limits for the proportion of urban buildings that could be built using bio-based materials have ranged from 90% [72] to 36% [71]. These differences largely arise from assumptions around harvesting practices and the extent to which afforestation takes place in the future; however it is worth noting that deforestation currently exceeds reforestation. The broader point underlying the differences between estimated values is that the potential future supply of structural bio-based materials is subject to numerous uncertainties that are largely beyond the control of the construction materials sector. Hence, bio-based materials represent a high-risk option to replace concrete at scale, which is ultimately not plausible within the critical timescale to 2050 [73]. Furthermore, bio-based materials are unsuitable for use in the majority of infrastructure applications give examples tunnels, dams, etc), for which they cannot replace concrete.

The overall contribution of increasing wood utilisation in construction to reduce the environmental impact of the sector is still debated. A recent study reports that although the substitution of steel and concrete with wood could save 0.8 to 0.9 Gt $CO_{2(eq.)}$ per year in steel and concrete emissions, these would be offset by wood harvesting increasing CO_2 emissions by about 0.7 to 0.9 Gt $CO_{2(eq.)}$ per year [43] associated with a projected increase in wood demand of about 1.8% per year [74]. Overall, the capacity for growth of the wood share in construction is limited. More importantly, a large proportion (50-100% depending on the modelled scenario in Peng et al. [43]) of the environmental benefits of concrete substitution by wood is offset by the associated increase in emissions of the harvesting sector.

A notable EU-level study on upscaling demonstrated that significantly enhancing the use of wood in construction could lead to an average annual reduction of 10 Mt $CO_{2(eq.)}$ per year, relative to a business-as-usual scenario [75]. This contrasts with the estimated carbon savings of reducing clinker factor in cement and optimising concrete formulation, with a potential to reduce emissions between 600-to-800 Mt of $CO_{2(eq.)}$ per year [76,77], a 60-to-80 fold increase over wood substitution benefits. Only an extreme (and likely unrealistic) scenario of wood production upscaling in EU to 1 m^3 wood per capita (currently 0.15 m^3/capita) could result in substitution benefits in the order of low carbon concrete technologies, around 600 Mt $CO_{2(eq.)}$ [78,79].

The well-established projection of Hildebrandt et al. [74] of a 1.8% annual increase of sawnwood production to 2030 defines a market level substitution impact of 88.7 Mt $CO_{2(eq.)}$ if 50% of the additional stock is used to replace concrete, steel and masonry in Europe [79]. This value considers the amount of waste and byproducts that are generated in the manufacture of sawnwood elements. However, it neglects the geographical mismatch between regions where a sustainable increase in wood production is possible compared to those where the actual surge in construction materials demand is. This value is in general agreement with CO_2 saving projections associated with an increase in wood utilisation in construction in Europe [75,78], with estimated values around 10 Mt $CO_{2(eq.)}$ per year of reduction.

The timber emissions in the base scenario have been set as 250 kg CO_2/t. timber (as described earlier in the Supplementary Methods). For the least favourable scenario, timber emissions have been set at 400 kg.CO_2 / t. timber: this corresponds to the highest value for A1-A3 emissions for CLT from 11 EPDs evaluated by IStructE [22]. The most favourable estimate uses a value of -200 kg.CO_2 / t. timber: this value has been derived using the methodology from [80], and results from assuming that the trees for timber used in construction have been replanted and grow taking up CO_2. In that work, Dunant & Allwood assumed that 55% of the tree mass ends up as timber, likely a favourable estimate (see [81]) but used the emissions of CLT for the CO_2 associated with production. As 2050 is less than 30 years in the future, and therefore less than the age at which e.g. Douglas Fir would be harvested, we believe this calculation is a generous estimate of the potential for carbon uptake of timber. Indeed, research on the global impacts of timber harvesting indicate that the emissions from the breakdown of waste biomass generated during harvesting are significant [43]. The range of timber production to 2050 we used was taken from the extrema of the FAO scenarios published in [82].

Building structural design

Dunant et al. observed that using software-aided optimisation of structural frames reduced the embodied carbon of structures [83]. This task is harder in the case of individual housing where the materials are much stronger than they need to be and the form of construction is itself wasteful in terms of useful habitable surface per amount of material used. For example, Drewniok et al. found that in the UK bungalows have 40-50% more embodied carbon per square metre than low rise residential construction [84].

It is debatable to what extent more efficient design can be applied in developing countries, taking into account the large part of construction (even up to 80-90%) which takes part in the informal sector in these countries. Given the very high relative cost of construction materials in these countries, it should not be assumed that the use of materials in these countries is as inefficient as in more developed countries where concrete especially is extremely cheap. For example, an average worker's daily wage could buy approximately twenty 50 kg bags of cement in Germany, but not a single bag in the Democratic Republic of Congo [85].

Reuse of concrete structural elements in new structures is another circular economy strategy which has the potential to displace production of primary concrete [86]. However, the feasibility of concrete reuse at present is mostly limited to pre-fabricated concrete elements [87]. As a result, the supply of concrete elements from end-of-life buildings is a major limiting factor for reuse; a study on Sweden, which has a mature building stock, found that even in recent years the inflows of pre-fabricated concrete elements were at least an order of magnitude greater than out flows [88]. In China, which has a less mature building stock, pre-fabricated buildings account for only 20% of new construction area [89]; even in the most restrictive scenario for new construction, authors estimated that only 3.8% of reinforced concrete could be reused in 2050.

Even within the limited available outflows, reuse of concrete elements is currently restricted by challenges around disassembly and validation; suitable testing procedures to guarantee structural performance are still in development [90]. Whilst the number of concrete reuse case studies is growing [91], the proportion of concrete reuse at the global concrete is negligible. Design-for-disassembly approaches can facilitate the reuse of concrete in future [92]; however, even if such approaches were adopted universally immediately, the benefits of reuse could only be realised at the end of structures' service life, which should be ≥50 years.

Due to the previous reasons stated, current evidence suggests that the carbon mitigation potential of concrete reuse will be highly limited over 2025-2050. For the study on Sweden previously cited, the potential emissions savings from reuse of all eligible elements were calculated to have a maximum saving of 1% of lifecycle emissions [88]. Thus, whilst concrete element reuse may be a valuable strategy to encourage over the longer term or in parts of the world where urban environments will rapidly be restructured, we do not include reuse within the group of strategies that we deem are technically feasible and readily implementable today at a global scale.

More efficient concrete production and extending the use of supplementary cementitious materials

Regarding the efficiency of concrete production, the baseline value for the cement content in concrete was set at 350 kg/m^3. As described in the main article, reducing even to a conservative cement content of 280 kg/m^3 (within EN-206 standard limits), the carbon footprint can be reduced about 20%. Regarding the use of supplementary cementitious materials, the baseline value for clinker substitution was set at 29%, which is the 2018 reported worldwide average value [93]. Based on recent developments using ternary cement blends including calcined clay and limestone, an average substitution level of 40% by 2030 and above 50% by 2050 was considered likely. Whilst these two strategies are physically distinct (the first acting on the concrete, second acting on the cement), they are widely deployed together.

For example, Supplementary Figure 4 shows the CO_2 emissions reported by the National Ready Mixed Concrete Association (NRMCA) of the U.S., and the average values currently observed in the UK. These values are compared with the low-carbon concrete benchmark established by the First Movers Coalition (FMC, managed by the World Economic Forum) and values observed for ready-mix concrete formulations incorporating natural pozzolans (73% clinker content in cement, binder content 240-350 kg/m^3) and LC^3-based concrete formulations produced in the laboratory (50% clinker content, binder content 230-300 kg/m^3). US mixes are routinely 30% more CO_2 intensive than European ones, which are in turn 30% more CO_2 intensive than Chilean ones, themselves again 30% more CO_2 intensive than well-optimised LC^3 blends. A significant fraction of these improvements is due to the use of SCMs.

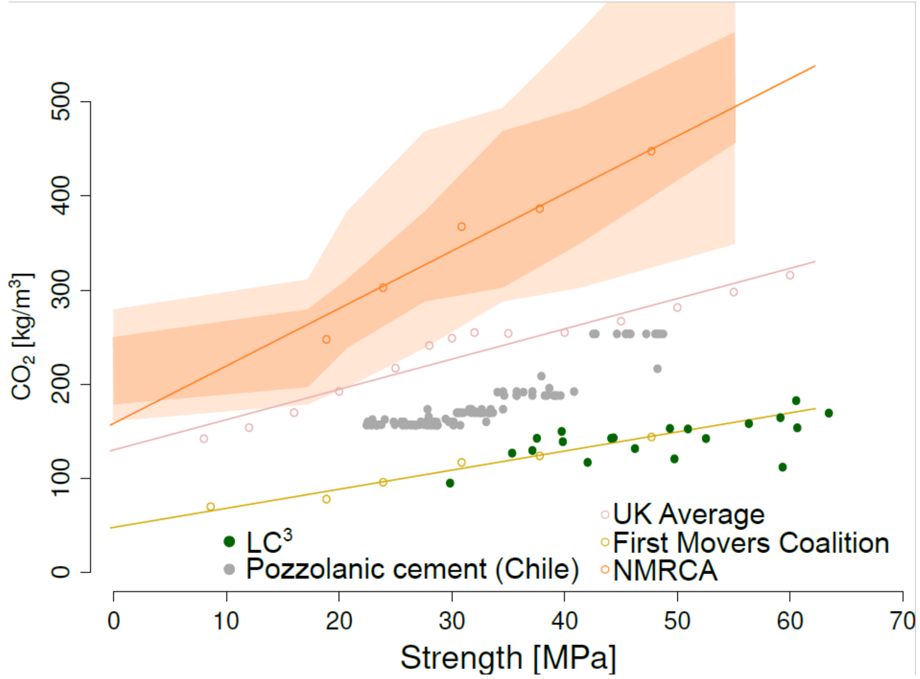

Supplementary Figure 4: Comparison of $CO_{2(eq.)}$ of current concrete formulations reported by the NRMCA (US) members [94] and UK average [95] with low-carbon concrete benchmarks by the First Movers Coalition (FMC) and the Low Carbon Concrete Group (LCCG) of the UK. A dataset of LC^3-based concrete formulations (lab-scale) and natural pozzolan (NP) based ready-mix concrete formulations from Chile (CL) [77] are also shown as comparison. For the NRMCA datapoints, the uncertainty bands have been calculated by taking the minimum and maximum strength in each class, as well as the minimum and maximum CO_2 reported. The deep colour band is produced from taking a minimum and a maximum each time, and the light colour by taking two minima or two maxima. The values used is provided in Supplementary Table 11.

Supplementary Table 11: Typical, minimum and maximum strength values for difference concretes, and their corresponding typical, minimum and maximum embodied CO_2 values. All data sourced from NRMCA.

**Minimum strength (MPa)**	**Maximum strength (MPa)**	**Typical strength (MPa)**	**Minimum CO_2 (kg/m^3)**	**Maximum CO_2 (kg/m^3)**	**Typical CO_2 (kg/m^3)**
0	17.2	8.6	178.72	279.56	223.52
17.2	20.7	18.9	197.09	311.39	247.73
20.7	27.6	23.9	238.71	383.6	302.60
27.6	34.5	30.9	288.1	468.89	367.54
34.5	41.4	37.8	302.75	493.67	386.60
41.3	55.1	47.7	349.15	574.1	447.71

Steel decarbonisation

Direct CO_2 emissions (i.e. Scope 1) in the EAF process arises from oxidation of small amounts of carbon in the steel scrap mix, cathode consumption and the use of small amounts of quicklime – a reasonable upper value is 40 kg $CO_{2(eq.)}$ / t.crude steel [96]. Electricity use for EAF

steel production using scrap is approximately 500 kWh/t [96]. Scope 2 emissions are highly dependent on the emissions factor of electricity used. For the <1.5°C scenario, IPCC AR6 projects that global electricity production will reach net-zero sometime between 2044 and 2055 [97]. In a worst case scenario of keeping only within the <2°C scenario, electricity emissions are still projected to reduce from 2020 levels (461 g.CO_2/kWh [98]) by at least 75% in 2050 [97], corresponding to a maximum of approximately 115 g.CO_2/kWh. Using this conservative value gives Scope 2 emissions of approximately 58 kg.CO_2 / t.crude steel. Summing the estimated Scope 1 and Scope 2 emissions described above gives a value of ~100 kg.CO_2 / t.crude steel (produced using scrap) in 2050, as used in the main article.

Circular use of concrete fines

From end-of-life concrete, both the aggregate and cement paste can be recycled to produce secondary concrete, with a range of processing routes developed for both material outflow streams. In this study, we investigate the use of concrete fines as a replacement for raw meal in clinker production as a decarbonisation strategy (i.e. "Circular use of concrete fines"). Dunant et al. [99] estimated that the available amount of concrete fines by 2050 globally will amount to approximately 60% of clinker consumption. This means that the reuse of only a quarter of these fines in kilns, using the process currently deployed by Holcim, could easily abate 10% of the clinker carbon intensity

We do not consider that other routes for use of recycled concrete fit the criteria of being technically feasible and readily implementable today at a global scale; we describe our reasons for excluding these other routes in the paragraphs below.

Recycled cement paste can be subjected to an accelerated carbonation treatment, which makes it suitable for use as an SCM – the subsequent replacement of clinker using the recycled cement paste SCM hence offers mitigation potential [100]. However, this strategy has so far only been demonstrated at the laboratory-scale using synthetic hydrated cement paste [100], so hence does not fulfil the criteria for being technically feasible and readily implementable today at a global scale.

Concrete at the end of life is commonly crushed and used as low quality aggregate. The use of recycled concrete aggregate is effective for reducing volumes of waste production and reducing raw material extraction. But due to the difficulty of completely removing hydrated cement paste from the surface of recycled concrete aggregate, more slightly water and cement is required for use of recycled concrete aggregates in concrete (for equivalent concrete strength class using natural aggregates); this results in a small increase in embodied carbon [101]. Whilst recycled concrete aggregate can offer modest carbon savings over natural aggregate in some specific situations, this is highly dependent on transport distances [101]. As a result, greater use of recycled concrete aggregate is not expected to lead to any meaningful carbon mitigation at the global scale, and so we have not considered it as a decarbonisation strategy.

REFERENCES

1 Hatfield, A. National Minerals Information Center: Cement Statistics and Information. (Reston, VA, 2021).

2 IEA. *Cement*, <https://www.iea.org/energy-system/industry/cement > (2023).

3 GCCA. Getting the numbers right (GNR) project. (London, UK, 2020).

4 IEA. Energy Technology Perspectives 2020: Special Report on Carbon Capture, Utilisation and Storage. (2020).

5 Olsson, J. A., Hafez, H., Miller, S. A. & Scrivener, K. L. Greenhouse Gas Emissions and Decarbonization Potential of Global Fired Clay Brick Production. *Environmental Science & Technology* **59**, 1909-1920 (2025). https://doi.org/10.1021/acs.est.4c08994

6 Ashby, M. F. Material Property Data for Engineering Materials. (ANSYS, Inc., Canonsburg, PA, 2021).

7 Westbroek, C. D., Bitting, J., Craglia, M., Azevedo, J. M. C. & Cullen, J. M. Global material flow analysis of glass: From raw materials to end of life. *Journal of Industrial Ecology* **25**, 333-343 (2021). https://doi.org/https://doi.org/10.1111/jiec.13112

8 IEA. Tracking Industrial Energy Efficiency and CO2 Emissions, International Energy Agency. (International Energy Agency, Paris, 2007).

9 United Nations Statistics Division. UN data: Bitumen. (2023).

10 Brown, E. R. Density of Asphalt Concrete - How Much is Needed? , (National Center for Asphalt Technology, Auburn, AL, 1990).

11 Shacat, J., Willis, J. R. & Ciavola, B. GHG emissions inventory for asphalt mix production in the United States. (National Asphalt Pavement Association, Greenbelt, MD, USA, 2022).

12 Tuck, C. National Minerals Information Center: Iron and Steel Statistics and Information. (United States Geological Survey, Reston, VA, 2021).

13 Cullen, J. M., Allwood, J. M. & Bambach, M. D. Mapping the Global Flow of Steel: From Steelmaking to End-Use Goods. *Environmental Science & Technology* **46**, 13048-13055 (2012). https://doi.org/10.1021/es302433p

14 IEA. *Steel*, <https://www.iea.org/energy-system/industry/steel> (2023).

15 IEA. Iron and Steel Technology Roadmap. (International Energy Agency, Paris, 2020).

16 Bray, E. National Minerals Information Center: Aluminum Statistics and Information. (Reston, VA, 2021).

17 Cullen, J. M. & Allwood, J. M. Mapping the Global Flow of Aluminum: From Liquid Aluminum to End-Use Goods. *Environmental Science & Technology* **47**, 3057-3064 (2013). https://doi.org/10.1021/es304256s

18 IEA. *Aluminium*, <https://www.iea.org/energy-system/industry/aluminium> (2023).

19 PlasticsEurope. Plastics - the Facts 2021. (PlasticsEurope, Brussels, Belgium, 2021).

20 Stegmann, P., Daioglou, V., Londo, M., van Vuuren, D. P. & Junginger, M. Plastic futures and their CO2 emissions. *Nature* **612**, 272-276 (2022). https://doi.org/10.1038/s41586-022-05422-5

21 UN FAOSTAT. *Forestry Production and Trade [WWW Document].* , <https://www.fao.org/faostat/en/#data/FO > (2023).

22 Place, T., Perkins, C. & Caine, L. Mass timber embodied carbon factors. (The Institution of Structural Engineers, London, UK, 2021).

23 Global Gypsum. *Plasterboard Focus 2030*, <https://globalgypsum.com/magazine/articles/802-plasterboard-focus-2030> (2022).

24 Knauf. Knauf Plasterboard Range. (Knauf, Sittingbourne, UK, 2025).
25 Circular Ecology. ICE (Inventory of Carbon & Energy) V4.0. (Circular Ecology, 2024).
26 Global Trade. *The global mineral wool market started to slow down*, <https://www.globaltrademag.com/the-global-mineral-wool-market-started-to-slow-down/> (2020).
27 Institut Bauen und Umwelt e.V. Environmental Product Declaration - DP7-DP-8 - Multipurpose Rock Mineral Wool insulation. (Berlin, 2018).
28 Hill, C., Norton, A. & Dibdiakova, J. A comparison of the environmental impacts of different categories of insulation materials. *Energy and Buildings* **162**, 12-20 (2018). https://doi.org/https://doi.org/10.1016/j.enbuild.2017.12.009
29 Scrivener, K. L., John, V. M. & Gartner, E. Eco-efficient cements: Potential economically viable solutions for a low-CO_2 cement-based materials industry. (UNEP, Paris, 2017).
30 Guo, R. *et al.* Global CO2 uptake by cement from 1930 to 2019. *Earth Syst. Sci. Data* **13**, 1791-1805 (2021). https://doi.org/10.5194/essd-13-1791-2021
31 Thamboo, J., Jayarathne, N. & Bandara, A. Characterisation and mix specification of commonly used masonry mortars. *SN Applied Sciences* **1**, 292 (2019). https://doi.org/10.1007/s42452-019-0312-z
32 Nisbet, M. A., Marceau, M. L. & VanGeem, M. G. Environmental life cycle inventory of portland cement concrete. (Portland Cement Association, 2002).
33 Ashby, M. F. in *Materials Selection in Mechanical Design (Fourth Edition)* (ed Michael F. Ashby) 495-523 (Butterworth-Heinemann, 2011).
34 Miller, S. A. & Moore, F. C. Climate and health damages from global concrete production. *Nature Climate Change* **10**, 439-443 (2020). https://doi.org/10.1038/s41558-020-0733-0
35 Pathak, M. *et al.* in *Climate Change 2022: Mitigation of Climate Change. Contribution of Working Group III to the Sixth Assessment Report of the Intergovernmental Panel on Climate Change* (eds P.R. Shukla *et al.*) (Cambridge University Press, 2022).
36 Van Roijen, E., Sethares, K., Kendall, A. & Miller, S. A. The climate benefits from cement carbonation are being overestimated. *Nature Communications* **15**, 4848 (2024). https://doi.org/10.1038/s41467-024-48965-z
37 Xi, F. *et al.* Substantial global carbon uptake by cement carbonation. *Nature Geoscience* **9**, 880-883 (2016). https://doi.org/10.1038/ngeo2840
38 Andersson, R., Fridh, K., Stripple, H. & Häglund, M. Calculating CO2 Uptake for Existing Concrete Structures during and after Service Life. *Environmental Science & Technology* **47**, 11625-11633 (2013). https://doi.org/10.1021/es401775w
39 Andrade, C. Evaluation of the degree of carbonation of concretes in three environments. *Construction and Building Materials* **230**, 116804 (2020). https://doi.org/https://doi.org/10.1016/j.conbuildmat.2019.116804
40 Cao, Z. *et al.* The sponge effect and carbon emission mitigation potentials of the global cement cycle. *Nature Communications* **11**, 3777 (2020). https://doi.org/10.1038/s41467-020-17583-w
41 Tibrewal, K. *et al.* Reconciliation of energy use disparities in brick production in India. *Nature Sustainability* **6**, 1248-1257 (2023). https://doi.org/10.1038/s41893-023-01165-x
42 Geyer, R., Jambeck, J. R. & Law, K. L. Production, use, and fate of all plastics ever made. *Science Advances* **3**, e1700782 (2017). https://doi.org/10.1126/sciadv.1700782

43 Peng, L., Searchinger, T. D., Zionts, J. & Waite, R. The carbon costs of global wood harvests. *Nature* **620**, 110-115 (2023). https://doi.org/10.1038/s41586-023-06187-1
44 Hawkins, W., Cooper, S., Allen, S., Roynon, J. & Ibell, T. Embodied carbon assessment using a dynamic climate model: Case-study comparison of a concrete, steel and timber building structure. *Structures* **33**, 90-98 (2021). https://doi.org/https://doi.org/10.1016/j.istruc.2020.12.013
45 Cabeza, L. F., Boquera, L., Chàfer, M. & Vérez, D. Embodied energy and embodied carbon of structural building materials: Worldwide progress and barriers through literature map analysis. *Energy and Buildings* **231**, 110612 (2021). https://doi.org/https://doi.org/10.1016/j.enbuild.2020.110612
46 Orr, J., Gibbons, O. & Arnold, W. How to calculate embodied carbon. (The Institution of Structural Engineers, 2020).
47 Guest, G., Bright, R. M., Cherubini, F. & Strømman, A. H. Consistent quantification of climate impacts due to biogenic carbon storage across a range of bio-product systems. *Environmental Impact Assessment Review* **43**, 21-30 (2013). https://doi.org/https://doi.org/10.1016/j.eiar.2013.05.002
48 Zhang, X., Chen, J., Dias, A. C. & Yang, H. Improving Carbon Stock Estimates for In-Use Harvested Wood Products by Linking Production and Consumption—A Global Case Study. *Environmental Science & Technology* **54**, 2565-2574 (2020). https://doi.org/10.1021/acs.est.9b05721
49 Al-Mejren, A. A. Credibility of growth and development measures in rentier economies: The case of GCC. *Journal of Economics and Development Studies* **7**, 83-100 (2019). https://doi.org/10.15640/jeds.v7n4a8
50 Soonsawad, N., Martinez, R. M. & Schandl, H. Material demand, and environmental and climate implications of Australia's building stock: Current status and outlook to 2060. *Resources, Conservation and Recycling* **180**, 106143 (2022). https://doi.org/https://doi.org/10.1016/j.resconrec.2021.106143
51 Arehart, J. H., Pomponi, F., D'Amico, B. & Srubar, W. V. Structural material demand and associated embodied carbon emissions of the United States building stock: 2020–2100. *Resources, Conservation and Recycling* **186**, 106583 (2022). https://doi.org/https://doi.org/10.1016/j.resconrec.2022.106583
52 Carcassi, O. B., Habert, G., Malighetti, L. E. & Pittau, F. Material Diets for Climate-Neutral Construction. *Environmental Science & Technology* **56**, 5213-5223 (2022). https://doi.org/10.1021/acs.est.1c05895
53 De Wolf, C. *et al.* Material quantities and embodied carbon dioxide in structures. *Proceedings of the Institution of Civil Engineers - Engineering Sustainability* **169**, 150-161 (2016). https://doi.org/10.1680/jensu.15.00033
54 El Hanandeh, A. Environmental assessment of popular single-family house construction alternatives in Jordan. *Building and Environment* **92**, 192-199 (2015). https://doi.org/https://doi.org/10.1016/j.buildenv.2015.04.032
55 Evangelista, P. P. A., Kiperstok, A., Torres, E. A. & Gonçalves, J. P. Environmental performance analysis of residential buildings in Brazil using life cycle assessment (LCA). *Construction and Building Materials* **169**, 748-761 (2018). https://doi.org/https://doi.org/10.1016/j.conbuildmat.2018.02.045
56 Heeren, N. & Fishman, T. A database seed for a community-driven material intensity research platform. *Scientific Data* **6**, 23 (2019). https://doi.org/10.1038/s41597-019-0021-x

57 Huang, T., Shi, F., Tanikawa, H., Fei, J. & Han, J. Materials demand and environmental impact of buildings construction and demolition in China based on dynamic material flow analysis. *Resources, Conservation and Recycling* **72**, 91-101 (2013). https://doi.org/https://doi.org/10.1016/j.resconrec.2012.12.013
58 Miatto, A. *et al.* A spatial analysis of material stock accumulation and demolition waste potential of buildings: A case study of Padua. *Resources, Conservation and Recycling* **142**, 245-256 (2019). https://doi.org/https://doi.org/10.1016/j.resconrec.2018.12.011
59 Mosteiro-Romero, M. *et al.* Relative importance of electricity sources and construction practices in residential buildings: A Swiss-US comparison of energy related life-cycle impacts. *Energy and Buildings* **68**, 620-631 (2014). https://doi.org/https://doi.org/10.1016/j.enbuild.2013.09.046
60 Reza, B., Sadiq, R. & Hewage, K. Emergy-based life cycle assessment (Em-LCA) of multi-unit and single-family residential buildings in Canada. *International Journal of Sustainable Built Environment* **3**, 207-224 (2014). https://doi.org/https://doi.org/10.1016/j.ijsbe.2014.09.001
61 Sprecher, B. *et al.* Material intensity database for the Dutch building stock: Towards Big Data in material stock analysis. *Journal of Industrial Ecology* **26**, 272-280 (2022). https://doi.org/https://doi.org/10.1111/jiec.13143
62 Marinova, S., Deetman, S., van der Voet, E. & Daioglou, V. Global construction materials database and stock analysis of residential buildings between 1970-2050. *Journal of Cleaner Production* **247**, 119146 (2020). https://doi.org/https://doi.org/10.1016/j.jclepro.2019.119146
63 van Engelenburg, M., Deetman, S., Fishman, T., Behrens, P. & van der Voet, E. TRIPI: A global dataset and codebase of the total resources in physical infrastructure encompassing road, rail, and parking. *Data in Brief* **54**, 110387 (2024). https://doi.org/https://doi.org/10.1016/j.dib.2024.110387
64 Hosseinijou, S. A. & Mansour, S. Dynamic Material Flow Analysis of Cement in Iran: New Insights for Sustainability of Civil Infrastructures. *Civil Engineering Infrastructures Journal* **54**, 381-403 (2021). https://doi.org/10.22059/ceij.2021.304150.1684
65 da Costa Reis, D., Mack-Vergara, Y. & John, V. M. Material flow analysis and material use efficiency of Brazil's mortar and concrete supply chain. *Journal of Industrial Ecology* **23**, 1396-1409 (2019). https://doi.org/https://doi.org/10.1111/jiec.12929
66 Wang, W. *et al.* A Material Flow Analysis (MFA)-based potential analysis of eco-efficiency indicators of China's cement and cement-based materials industry. *Journal of Cleaner Production* **112**, 787-796 (2016). https://doi.org/https://doi.org/10.1016/j.jclepro.2015.06.103
67 Manning, D. A. C., Tangtinthai, N. & Heidrich, O. Evaluation of raw material extraction, processing, construction and disposal of cement and concrete products: datasets and calculations. *Data in Brief* **24**, 103929 (2019). https://doi.org/https://doi.org/10.1016/j.dib.2019.103929
68 Watari, T., Cao, Z., Hata, S. & Nansai, K. Efficient use of cement and concrete to reduce reliance on supply-side technologies for net-zero emissions. *Nature Communications* **13**, 4158 (2022). https://doi.org/10.1038/s41467-022-31806-2
69 Watari, T., Cabrera Serrenho, A., Gast, L., Cullen, J. & Allwood, J. Feasible supply of steel and cement within a carbon budget is likely to fall short of expected global

demand. *Nature Communications* **14**, 7895 (2023). https://doi.org/10.1038/s41467-023-43684-3
70 Marsh, A., Dillon, T. & Bernal, S. Cement and concrete decarbonisation roadmaps – a meta-analysis within the context of the United Kingdom. *RILEM Technical Letters* **8**, 94-105 (2023). https://doi.org/10.21809/rilemtechlett.2023.163
71 Pomponi, F., Hart, J., Arehart, J. H. & D'Amico, B. Buildings as a Global Carbon Sink? A Reality Check on Feasibility Limits. *One Earth* **3**, 157-161 (2020). https://doi.org/https://doi.org/10.1016/j.oneear.2020.07.018
72 Churkina, G. *et al.* Buildings as a global carbon sink. *Nature Sustainability* **3**, 269-276 (2020). https://doi.org/10.1038/s41893-019-0462-4
73 Hafez, H., Marsh, A. T. M., Flagar, M., Peng, L. & Scrivener, K. L. Concrete is a lower carbon option than engineered bio-based materials to meet global urban housing demand. (under review).
74 Hildebrandt, J., Hagemann, N. & Thrän, D. The contribution of wood-based construction materials for leveraging a low carbon building sector in europe. *Sustainable Cities and Society* **34**, 405-418 (2017). https://doi.org/https://doi.org/10.1016/j.scs.2017.06.013
75 S. Rüter, F. W., N. Forsell. Climate benefits of material substitution by forest biomass and harvested wood products: Perspective 2030 - Final Report. (Johann Heinrich von Thünen-Institut, 2016).
76 Zunino, F., Martirena, F. & Scrivener, K. Limestone Calcined Clay Cements (LC3). *ACI Materials Journal* **118** (2021). https://doi.org/10.14359/51730422
77 Zunino, F. A two-fold strategy towards low-carbon concrete. *RILEM Technical Letters* **8**, 45-58 (2023). https://doi.org/10.21809/rilemtechlett.2023.179
78 Eriksson, L. O. *et al.* Climate change mitigation through increased wood use in the European construction sector—towards an integrated modelling framework. *European Journal of Forest Research* **131**, 131-144 (2012). https://doi.org/10.1007/s10342-010-0463-3
79 Leskinen, P. *et al.* Substitution effects of wood-based products in climate change mitigation. (European Forest Institute, 2018).
80 Dunant, C. F. & Allwood, J. M. What investments in material production are needed to achieve net-zero construction in the UK by 2050? *Journal of Cleaner Production* **464**, 142709 (2024). https://doi.org/https://doi.org/10.1016/j.jclepro.2024.142709
81 Matthews, R., Jenkins, T., Mackie, E. & Dick, E. Forest Yield: A handbook on forest growth and yield tables for British forestry. (Forestry Commission, 2016).
82 Branthomme, A. *et al.* in *The State of the World's Forests 2024 – Forest-sector innovations towards a more sustainable future* (eds Wu Zhimin *et al.*) (FAO, 2024).
83 Dunant, C. F., Drewniok, M. P., Eleftheriadis, S., Cullen, J. M. & Allwood, J. M. Regularity and optimisation practice in steel structural frames in real design cases. *Resources, Conservation and Recycling* **134**, 294-302 (2018). https://doi.org/https://doi.org/10.1016/j.resconrec.2018.01.009
84 Drewniok, M. P., Dunant, C. F., Allwood, J. M., Ibell, T. & Hawkins, W. Modelling the embodied carbon cost of UK domestic building construction: Today to 2050. *Ecological Economics* **205**, 107725 (2023). https://doi.org/https://doi.org/10.1016/j.ecolecon.2022.107725

85 Mwiti Marangu, J. *et al.* Five recommendations to accelerate sustainable solutions in cement and concrete through partnership. *RILEM Technical Letters* **8**, 1-11 (2023). https://doi.org/10.21809/rilemtechlett.2023.173
86 Marsh, A. T. M., Velenturf, A. P. M. & Bernal, S. A. Circular Economy strategies for concrete: implementation and integration. *Journal of Cleaner Production* **362**, 132486 (2022). https://doi.org/https://doi.org/10.1016/j.jclepro.2022.132486
87 Ostapska, K., Rüther, P., Loli, A. & Gradeci, K. Design for Disassembly: A systematic scoping review and analysis of built structures Designed for Disassembly. *Sustainable Production and Consumption* **48**, 377-395 (2024). https://doi.org/https://doi.org/10.1016/j.spc.2024.05.014
88 Al-Najjar, A., Malmqvist, T., Stenberg, E. & Höjer, M. Stock, flow and reuse potential of precast concrete in Swedish residential buildings: Embodied carbon assessment. *Resources, Conservation and Recycling* **218**, 108229 (2025). https://doi.org/https://doi.org/10.1016/j.resconrec.2025.108229
89 Zhu, C., Li, X., Zhu, W. & Gong, W. Embodied carbon emissions and mitigation potential in China's building sector: An outlook to 2060. *Energy Policy* **170**, 113222 (2022). https://doi.org/https://doi.org/10.1016/j.enpol.2022.113222
90 Devènes, J., Bastien-Masse, M. & Fivet, C. Reusability assessment of reinforced concrete components prior to deconstruction from obsolete buildings. *Journal of Building Engineering* **84**, 108584 (2024). https://doi.org/https://doi.org/10.1016/j.jobe.2024.108584
91 Küpfer, C., Bastien-Masse, M. & Fivet, C. Reuse of concrete components in new construction projects: Critical review of 77 circular precedents. *Journal of Cleaner Production* **383**, 135235 (2023). https://doi.org/https://doi.org/10.1016/j.jclepro.2022.135235
92 Salama, W. Design of concrete buildings for disassembly: An explorative review. *International Journal of Sustainable Built Environment* **6**, 617-635 (2017). https://doi.org/https://doi.org/10.1016/j.ijsbe.2017.03.005
93 Andrew, R. M. Global CO2 emissions from cement production, 1928–2018. *Earth Syst. Sci. Data* **11**, 1675-1710 (2019). https://doi.org/10.5194/essd-11-1675-2019
94 NRMCA. A Cradle-to-Gate Life Cycle Assessment of Ready-Mixed Concrete Manufactured by NRMCA Members – Version 3.2. (National Ready-Mix Concrete Association 2022).
95 LCCG. The UK Low Carbon Concrete Group Market Benchmark. (The UK Low Carbon Concrete Group, 2023).
96 Yang, L. *et al.* Life cycle carbon footprint of electric arc furnace steelmaking processes under different smelting modes in China. *Sustainable Materials and Technologies* **35**, e00564 (2023). https://doi.org/https://doi.org/10.1016/j.susmat.2022.e00564
97 Clarke, L., Y.-M. Wei, A. De La Vega Navarro, A. Garg, A.N. Hahmann, S. Khennas, I.M.L. Azevedo, A. Löschel, A.K. Singh, L. Steg, G. Strbac, K. Wada. in *Climate Change 2022: Mitigation of Climate Change. Contribution of Working Group III to the Sixth Assessment Report of the Intergovernmental Panel on Climate Change* (ed J. Skea P.R. Shukla, R. Slade, A. Al Khourdajie, R. van Diemen, D. McCollum, M. Pathak, S. Some, P. Vyas, R. Fradera, M. Belkacemi, A. Hasija, G. Lisboa, S. Luz, J. Malley) Ch. 6, (Cambridge University Press, 2022).
98 IEA. Electricity 2025. (International Energy Agency, Paris, 2025).

99 Dunant, C. F., Joseph, S., Prajapati, R. & Allwood, J. M. Electric recycling of Portland cement at scale. *Nature* **629**, 1055-1061 (2024). https://doi.org/10.1038/s41586-024-07338-8

100 Zajac, M., Skocek, J., Gołek, Ł. & Deja, J. Supplementary cementitious materials based on recycled concrete paste. *Journal of Cleaner Production* **387**, 135743 (2023). https://doi.org/https://doi.org/10.1016/j.jclepro.2022.135743

101 Marinković, S., Radonjanin, V., Malešev, M. & Ignjatović, I. Comparative environmental assessment of natural and recycled aggregate concrete. *Waste Management* **30**, 2255-2264 (2010). https://doi.org/https://doi.org/10.1016/j.wasman.2010.04.012
