# Supplementary material for: Timely deployment of best-in-class technologies to enable development and decarbonise construction
Source: Nat Commun. 2025 Dec 24;17:799. doi: 10.1038/s41467-025-67489-8 (PMC12824142; doi:10.1038/s41467-025-67489-8)
Supplement: Supplementary file 2 — Reporting Summary [file 41467_2025_67489_MOESM2_ESM.pdf]

nature portfolio

Corresponding author(s): Karen L. Scrivener

Last updated by author(s): Nov 14, 2025

Reporting Summary

Nature Portfolio wishes to improve the reproducibility of the work that we publish. This form provides structure for consistency and transparency in reporting. For further information on Nature Portfolio policies, see our Editorial Policies and the Editorial Policy Checklist.

Statistics

For all statistical analyses, confirm that the following items are present in the figure legend, table legend, main text, or Methods section.

n/a	Confirmed	
[ ]	[x]	The exact sample size (*n*) for each experimental group/condition, given as a discrete number and unit of measurement
[x]	[ ]	A statement on whether measurements were taken from distinct samples or whether the same sample was measured repeatedly
[x]	[ ]	The statistical test(s) used AND whether they are one- or two-sided<br>*Only common tests should be described solely by name; describe more complex techniques in the Methods section.*
[x]	[ ]	A description of all covariates tested
[x]	[ ]	A description of any assumptions or corrections, such as tests of normality and adjustment for multiple comparisons
[ ]	[x]	A full description of the statistical parameters including central tendency (e.g. means) or other basic estimates (e.g. regression coefficient) AND variation (e.g. standard deviation) or associated estimates of uncertainty (e.g. confidence intervals)
[x]	[ ]	For null hypothesis testing, the test statistic (e.g. *F*, *t*, *r*) with confidence intervals, effect sizes, degrees of freedom and *P* value noted<br>*Give P values as exact values whenever suitable.*
[x]	[ ]	For Bayesian analysis, information on the choice of priors and Markov chain Monte Carlo settings
[x]	[ ]	For hierarchical and complex designs, identification of the appropriate level for tests and full reporting of outcomes
[x]	[ ]	Estimates of effect sizes (e.g. Cohen's *d*, Pearson's *r*), indicating how they were calculated

Our web collection on statistics for biologists contains articles on many of the points above.

Software and code

Policy information about availability of computer code

Data collection	No software was used for data collection.
Data analysis	Data analysis was carried out using Microsoft Excel 2021 and R.

For manuscripts utilizing custom algorithms or software that are central to the research but not yet described in published literature, software must be made available to editors and reviewers. We strongly encourage code deposition in a community repository (e.g. GitHub). See the Nature Portfolio guidelines for submitting code & software for further information.

Data

Policy information about availability of data

All manuscripts must include a data availability statement. This statement should provide the following information, where applicable:

- Accession codes, unique identifiers, or web links for publicly available datasets
- A description of any restrictions on data availability
- For clinical datasets or third party data, please ensure that the statement adheres to our policy

The data used in this study are available in the Zenodo database under accession code https://doi.org/10.5281/zenodo.17604460 . The data underpinning the projection assumptions used in this study are provided in the Supplementary Information.

Research involving human participants, their data, or biological material

Policy information about studies with human participants or human data. See also policy information about sex, gender (identity/presentation), and sexual orientation and race, ethnicity and racism.

Reporting on sex and gender	n/a
Reporting on race, ethnicity, or other socially relevant groupings	n/a
Population characteristics	n/a
Recruitment	n/a
Ethics oversight	n/a

Note that full information on the approval of the study protocol must also be provided in the manuscript.

Field-specific reporting

Please select the one below that is the best fit for your research. If you are not sure, read the appropriate sections before making your selection.

☐ Life sciences ☐ Behavioural & social sciences ☒ Ecological, evolutionary & environmental sciences

For a reference copy of the document with all sections, see nature.com/documents/nr-reporting-summary-flat.pdf

Ecological, evolutionary & environmental sciences study design

All studies must disclose on these points even when the disclosure is negative.

Study description	This study performed a new analysis on existing data (national wealth and material production), to develop new worldwide projections for future material demand, and analyse the effect on net CO2 emissions of applying various mitigation strategies. No primary data was generated, and the study was solely desk-based with no experimental, clinical or field work undertaken.
Research sample	The research sample was the countries of the world; the research data was national economic and material production data.
Sampling strategy	A sampling strategy was not relevant for this study; the population in question was national economic and material production data, which was already available in existing datasets. The datasets used are described in the Methods and Supplementary Methods.
Data collection	Data was collected from existing datasets, as described in the Methods and Supplementary Methods. This study analysed existing datasets in a new way; no new, primary data was collected.
Timing and spatial scale	Data was collected by the authors from existing databases, over the time period of 2023-2025.
Data exclusions	As described in the Supplementary Methods (and listed in Supplementary Table 3), certain outlier countries were excluded from the dataset for the top-down analysis. The purpose of this was to obtain master curves that were representative of the global trends, and certain atypical countries were excluded in order to avoid skewing the dataset. These included island nations and petro-states with atypical economic characteristics. Oil-exporting countries (Kuwait, Oman, Saudi Arabia, UAE and Qatar) were outliers, due to their large transient populations, atypical consumption patterns and the large contribution of hydrocarbons to their GDP (>40%).
Reproducibility	Not applicable, as no experimental or clinical work was carried out.
Randomization	Not applicable, as no experimental or clinical work was carried out.
Blinding	Not applicable, as no experimental or clinical work was carried out.

Did the study involve field work? ☐ Yes ☒ No

Reporting for specific materials, systems and methods

We require information from authors about some types of materials, experimental systems and methods used in many studies. Here, indicate whether each material, system or method listed is relevant to your study. If you are not sure if a list item applies to your research, read the appropriate section before selecting a response.

Materials & experimental systems

n/a	Involved in the study
☒	☐ Antibodies
☒	☐ Eukaryotic cell lines
☒	☐ Palaeontology and archaeology
☒	☐ Animals and other organisms
☒	☐ Clinical data
☒	☐ Dual use research of concern
☒	☐ Plants

Methods

n/a	Involved in the study
☒	☐ ChIP-seq
☒	☐ Flow cytometry
☒	☐ MRI-based neuroimaging

Plants

Seed stocks	n/a
Novel plant genotypes	n/a
Authentication	n/a
